# Differences in hippocampal subfield volume are seen in phenotypic variants of early onset Alzheimer's disease

**DOI:** 10.1016/j.nicl.2018.101632

**Published:** 2018-12-11

**Authors:** Thomas D. Parker, Catherine F. Slattery, Keir X.X. Yong, Jennifer M. Nicholas, Ross W. Paterson, Alexander J.M. Foulkes, Ian B. Malone, David L. Thomas, David M. Cash, Sebastian J. Crutch, Nick C. Fox, Jonathan M. Schott

**Affiliations:** a Department of Neurodegenerative Disease, Queen Square Institute of Neurology, UCL, London, UK; b Department of Medical Statistics, London School of Hygiene and Tropical Medicine, London, UK; c Neuroradiological Academic Unit, Department of Brain Repair and Rehabilitation, UCL Institute of Neurology, London, UK; d Leonard Wolfson Experimental Neurology Centre, UCL Institute of Neurology, London, UK

**Keywords:** Early-onset Alzheimer's disease, Posterior cortical atrophy, Hippocampal subfields, CA1, Presubiculum, Atypical Alzheimer's disease, Aβ, Beta-amyloid, CA, Cornu ammonis, CSF, Cerebrospinal fluid, EOAD, Early onset Alzheimer's disease, GCMLDG, Molecular and granule cell layers of the dentate gyrus, GDA, Graded difficulty arithmetic, GDST, Graded difficulty spelling test, HATA, Hippocampal amygdala transition area, LOAD, late onset Alzheimer's disease, MMSE, Mini-mental state examination, MPRAGE, Magnetization-prepared rapid gradient-echo, MRI, Magnetic resonance imaging, NART, National adult reading test, PCA, Posterior cortical atrophy, PCA-AD, PCA attributable to Alzheimer's disease, sRMT, Short recognition memory test, SPM, Statistical parametric mapping, tAD, Typical Alzheimer's disease, TE/TI/TR, Echo time/inversion time/repetition time, TIV, Total intracranial volume, VOSP, Visual object and space perception battery, WASI, Wechsler abbreviated scale of intelligence

## Abstract

The most common presentation of early onset Alzheimer's disease (EOAD – defined as symptom onset <65 years) is with progressive episodic memory impairment – amnestic or typical Alzheimer's disease (tAD). However, EOAD is notable for its phenotypic heterogeneity, with posterior cortical atrophy (PCA) – characterised by prominent higher-order visual processing deficits and relative sparing of episodic memory – the second most common canonical phenotype. The hippocampus, which comprises a number of interconnected anatomically and functionally distinct subfields, is centrally involved in Alzheimer's disease and is a crucial mediator of episodic memory. The extent to which volumes of individual hippocampal subfields differ between different phenotypes in EOAD is unclear. The aim of this analysis was to investigate the hypothesis that patients with a PCA phenotype will exhibit differences in specific hippocampal subfield volumes compared to tAD. We studied 63 participants with volumetric T1-weighted MRI performed on the same 3T scanner: 39 EOAD patients [27 with tAD and 12 with PCA] and 24 age-matched controls. Volumetric estimates of the following hippocampal subfields for each participant were obtained using Freesurfer version 6.0: CA1, CA2/3, CA4, presubiculum, subiculum, hippocampal tail, parasubiculum, the molecular and granule cell layers of the dentate gryus (GCMLDG), the molecular layer, and the hippocampal amygdala transition area (HATA). Linear regression analyses comparing mean hippocampal subfield volumes between groups, adjusting for age, sex and head size, were performed. Using a Bonferonni-corrected *p*-value of *p* < 0.0025, compared to controls, tAD was associated with atrophy in all hippocampal regions, except the parasubiculum. In PCA patients compared to controls, the strongest evidence for volume loss was in the left presubiclum, right subiculum, right GCMLDG, right molecular layer and the right HATA. Compared to PCA, patients with tAD had strong evidence for smaller volumes in left CA1 and left hippocampal tail. In conclusion, these data provide evidence that hippocampal subfield volumes differ in different phenotypes of EOAD.

## Highlights

• Early onset Alzheimer's disease (EOAD) is notable for its phenotypic heterogeneity.
• Data from a cohort of patients with EOAD and age-matched controls were analysed.
• Estimates of hippocampal subfield volumes were obtained using Freesurfer 6.0.
• Patients with amnestic and non-amnestic visual-led phenotypes of EOAD were compared.
• Visual-led phenotypes were associated with relative sparing of subfield atrophy.

## 1 Introduction

Early onset Alzheimer's disease (EOAD – defined as symptom onset under the age of 65) represents the most common cause of young onset dementia (Harvey et al., 2003) and often poses a significant diagnostic challenge (Rossor et al., 2010). As is the case in the more common late-onset form of Alzheimer's disease (LOAD), the most common presentation of EOAD is the amnestic led typical form of Alzheimer's disease (tAD), characterised by progressive episodic memory impairment. However, compared to LOAD, a higher proportion of EOAD patients present with non-amnestic atypical phenotypes (Mendez, 2012; Rossor et al., 2010; Slattery et al., 2017). The most commonly encountered atypical phenotype is that of posterior cortical atrophy (PCA), which is characterised by prominent higher-order visual processing deficits and relative sparing of episodic memory (Crutch et al., 2012, 2017). Why individuals with EOAD are more likely to develop these often markedly different phenotypes, is not clear.

*In vivo* brain imaging has provided significant insights into pathophysiological differences between tAD and PCA (Alves et al., 2013). Whereas deposition of beta-amyloid (Aβ) occurs throughout the cortex and does not show major differences between tAD and PCA phenotypes (Lehmann et al., 2013; Ossenkoppele et al., 2016; Whitwell et al., 2018), marked differences in cortical grey matter volume/cortical thickness (Lehmann et al., 2011; Poulakis et al., 2018; Ridgway et al., 2012; Whitwell et al., 2018; Whitwell et al., 2007), glucose hypometabolism (Lehmann et al., 2013; Whitwell et al., 2018), cerebral blood flow (Lehmann et al., 2016) and tau positron emission tomography tracer uptake (Ossenkoppele et al., 2016; Whitwell et al., 2018), most notably in the posterior parietal and occipital cortices, have been observed between tAD and PCA phenotypes.

In addition to brain imaging changes apparent in the cortex, there is also interest in how differences in subcortical structures may relate to phenotypic heterogeneity. The hippocampus is centrally involved in Alzheimer's disease, and although it is not the only brain region implicated (Aggleton et al., 2016), it plays a central role in mediating episodic memory (Dickerson and Eichenbaum, 2010). Lower total hippocampal volume measured *in vivo* is associated with clinically detectable Alzheimer's disease (Jack et al., 1992, 2000; Kesslak et al., 1991; Scheltens et al., 1992; Seab et al., 1988), is predictive of neuropathological features of Alzheimer's at post-mortem (Bobinski et al., 2000; Jack et al., 2002), and has been incorporated into contemporary diagnostic criteria for Alzheimer's disease (Dubois et al., 2014; McKhann et al., 2011). In patients with PCA, total hippocampal volume has been shown to be reduced compared to controls, although to a much lesser extent than the volume loss observed in tAD (Manning et al., 2015; Peng et al., 2016). Differences in hippocampal morphology between tAD and PCA patients, thought to reflect the relative preservation of hippocampal tissue in PCA (Manning et al., 2015), have also been reported.

However, the hippocampus is not a homogenous structure, and comprises a number of interconnected anatomically distinct subfields (Small et al., 2011). Neuropathological studies suggest differential pathological changes occur between hippocampal subfields in Alzheimer's disease. Neurofibrillary tangles, for example, are initially deposited in CA1, then the subiculum, CA2, CA3, CA4 and dentate gyrus (Braak and Braak, 1991; Lace et al., 2009). In keeping with this, Alzheimer's disease-related changes in grey matter microstructure (e.g. neuronal/dendritic loss) have also been shown to be most prominent in CA1 (Akram et al., 2008; Kerchner et al., 2014; Price et al., 2001; Rössler et al., 2002; Scheff et al., 2007; West et al., 1994). Although technically challenging, there has been considerable interest in quantifying the volumes of different hippocampal subfields *in vivo* using structural MRI and there is evidence to suggest preferential atrophy of certain subfields, especially CA1 and the presubiculum/subiculum complex in tAD (Apostolova et al., 2010, Apostolova et al., 2006; Blanken et al., 2017; Carlesimo et al., 2015; Iglesias et al., 2015; La Joie et al., 2013; Mak et al., 2017; Mueller et al., 2010; Pini et al., 2016; Wisse et al., 2014a, Wisse et al., 2014b).

In addition to being anatomically distinct, there is evidence to suggest that hippocampal subfields are functionally distinct (Small et al., 2011). CA1 in particular has been implicated as having particularly prominent role in sub-components of episodic memory (Bartsch et al., 2011; Bartsch et al., 2010; Dimsdale-Zucker et al., 2018).

To what extent the volume of individual hippocampal subfields differ between contrasting phenotypes in EOAD is unclear. Given the relative sparing of episodic memory in PCA compared to tAD (Crutch et al., 2017; Crutch et al., 2012); the relative sparing of total hippocampal atrophy in PCA compared to tAD (Manning et al., 2015; Peng et al., 2016); and evidence from healthy adults that individual subfields are implicated in sub-components of episodic memory (Bartsch et al., 2011, Bartsch et al., 2010; Dimsdale-Zucker et al., 2018), we hypothesised that there would be differences in specific hippocampal subfield volumes between patients with a PCA phenotype and those with tAD. In particular, we hypothesised that patients with tAD would have more atrophy in sub-fields most clearly implicated in episodic memory (e.g. CA1), and that these would be relatively spared in PCA; and that given that higher order visual problems are associated with right sided cortical atrophy, that compared to each other, tAD would show more left and PCA more right-sided subfield loss. With this in mind the aim of this study was to investigate the extent of atrophy of specific hippocampal subfields in EOAD using data from a population of patients with both tAD and PCA phenotypes, as well as age-matched healthy controls.

## 2 Methods

### 2.1 Participants

A total of 45 patients meeting consensus criteria for probable Alzheimer's disease (McKhann et al., 2011) with symptom onset <65 years were recruited prospectively from 2013 to 2015 from a specialist cognitive disorders clinic (Parker et al., 2018; Slattery et al., 2017). Documentation of the age at symptom onset and the presenting cognitive symptom were recorded for all patients. Patients included in the analysis were classified as having an amnestic (McKhann et al., 2011) or PCA (Crutch et al., 2017; Tang-Wai et al., 2004) phenotype according to published criteria. Cerebrospinal fluid (CSF) neurodegenerative markers were available for 34/39 patients (25/27 tAD and 9/12 PCA patients). All patients with CSF available had profiles consistent with Alzheimer pathology (mean Aβ1–42 = 404 ± 52 ng/l; tau:Aβ1–42 ratio = 2.20 ± 1.41) (Weston et al., 2015), therefore fulfilling NIA and IWG-2 criteria for AD (Dubois et al., 2014; McKhann et al., 2011), and in the case of the PCA group fulfilling the criteria for PCA attributable to Alzheimer's disease (PCA-AD) (Crutch et al., 2017). No patients had prominent pyramidal/extrapyramidal motor signs or visual hallucinations to suggest underlying cortico-basal degeneration or Lewy body pathology. No individual scored >4 on the Hachinski Ischaemic Score making a vascular aetiology unlikely (Moroney et al., 1997; Slattery et al., 2017). Twenty-four participants with no history of cognitive concerns were recruited as healthy controls matched for age and sex and were predominantly spouses of the EOAD patients. Detailed multi-domain cognitive testing was performed for each participant including: the mini-mental state examination (MMSE (Folstein et al., 1975)); an assessment of general intellect (vocabulary and matrices subtests of the Wechsler Abbreviated Scale of Intelligence (WASI) (Wechsler, 1999)); digit span forwards and backwards (Wechsler, 1987); episodic memory for faces and words (Short Recognition Memory Test (sRMT) (Warrington, 1984)); letter and category fluency; numeracy (Graded Difficulty Arithmetic (GDA) (Jackson and Warrington, 1986)); spelling (Graded Difficulty Spelling Test (GDST) (Baxter and Warrington, 1994)); the National Adult Reading Test (NART) (Nelson, 1982); visual search (letter (‘A’) cancellation) (Willison and Warrington, 1992); and the visual object and space perception (VOSP) battery (Warrington and James, 1991), which included shape detection (early visual processing), fragmented letters (visuoperception), object decision (visuoperception) and dot-counting (visuospatial processing). Ethical approval was obtained from the National Hospital for Neurology and Neurosurgery Research Ethics Committee and written informed consent was obtained from all the participants.

#### 2.1.1 APOE genotyping

Patient participants gave separate specific consent to donate blood for genetic analyses. DNA was extracted and *APOE* genotype was determined by PCR with 3′-minor groove binding probes (Slattery et al., 2017). *APOE* genotype data was not available for healthy control participants.

### 2.2 Image acquisition

All participants were scanned on the same Siemens Magnetom Trio (Siemens, Erlangen, Germany) 3T MRI scanner using a 32-channel phased array receiver head coil. Sagittal 3D MPRAGE T1-weighted volumetric MRI sequence (TE/TI/TR = 2.9/900/2200 ms, matrix size 256 × 256 × 208, voxel size 1.1 × 1.1 × 1.1 mm^3^) were performed for each participant.

### 2.3 Hippocampal subfield volume estimation

**Fig. 1:**
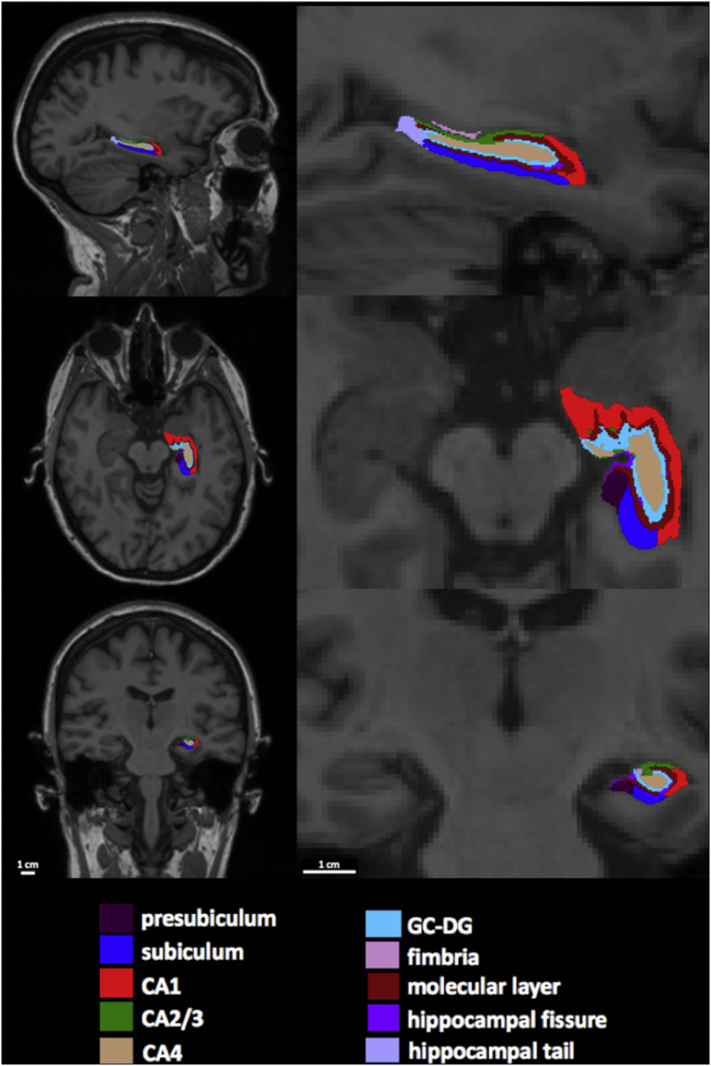
Example of segmentation of the left hippocampal formation into constituent subfields in the sagittal, axial, and coronal planes (NB. selected slices do not show the relatively smaller hippocampal amygdala transition area and parasubiculum).

Volumetric estimates of hippocampal formation subfields were performed using Freesurfer version 6.0. This algorithm is based on a computational atlas of the hippocampal formation using ex vivo, ultra-high resolution MRI and includes: CA1, CA2/3, CA4, fimbria, the hippocampal fissure, presubiculum, subiculum, hippocampal tail, parasubiculum, the molecular and granule cell layers of the dentate gyrus (GCMLDG), the molecular layer and the hippocampal amygdala transition area (HATA) (Iglesias et al., 2015). We did not include the hippocampal fissure, which is a thin CSF layer rather than a hippocampal substructure per se, nor did we include the fimbria, which is a small volume white matter region. It is also important to note that the hippocampal tail is not a histologically distinct region*,* but instead represents a conglomeration of CA1–4 and dentate gyrus, which are indistinguishable at this resolution due to the posterior narrowing. The hippocampal subfield segmentation and corresponding T1-weighted structural images for each participant were visually inspected using Freesurfer's Freeview (see Fig. 1). This was performed with the caveat that the limited spatial resolution provided by 3T MRI means precise visualisation of the boundaries that define the distinct hippocampal subfields is not possible at this field strength. Total intracranial volume (TIV) was calculated using statistical parametric mapping (SPM) software (SPM12; http://www.fil.ion.ucl.ac.uk/spm) (Malone et al., 2015).

### 2.4 Exclusions

One patient with tAD was found to have an autosomal dominant (Presenilin 1) mutation (Beck et al., 2014) and was excluded from the analysis based on the fact that familial Alzheimer's disease may be considered a distinct entity (Ryan et al., 2016). EOAD patients with language-led or behavioural phenotypes were not included in the analysis due to limited numbers (*n* = 2 and *n* = 1 respectively). One PCA patient failed Freesurfer processing and one PCA participant was excluded on the basis of severe motion artefact degrading tissue contrast in the hippocampus. Therefore, we included 39 EOAD patients in this analysis.

### 2.5 Statistical analysis

Demographics, clinical characteristics and performance on neuropsychology testing were compared between each group. For continuous characteristics, a Wilcoxon rank sum test was used, while categorical characteristics were compared between groups using Fisher's exact test. To investigate group differences in hippocampal subfield volumes, linear regression analyses comparing mean hippocampal subfield volumes between groups after adjustment for age, sex and TIV were performed. To correct for the multiple comparisons resulting from investigating both the left and right volumes of 10 different hippocampal subfields, a threshold of *p* < 0.0025 for formal statistical significance was used after Bonferroni correction for comparison across the 20 regions of interest. To aid in comparison of effect sizes between subfields, adjusted differences between YOAD patients and controls were expressed as a percentage change of the unadjusted mean volume for healthy controls. Adjusted differences between tAD patients and PCA patients were presented as a percentage change of the unadjusted mean volume for PCA patients. To investigate whether *APOE* genotype influenced hippocampal subfields a supplementary analysis comparing subfield volumes between *APOE* ε4 carriers and APOE ε4 non-carriers with and without adjustment for phenotype was performed.

## 3 Results

### 3.1 Participant demographic, clinical and neuropsychological data

Basic demographic, clinical and neuropsychological data and comparison by phenotype is summarised in Table 1. There were no significant differences in age, sex, handedness or years of education when comparing groups. There were no significant differences in MMSE score, disease duration or proportion of *APOE* ε4 carriers when comparing tAD and PCA patients. As expected, performance on all cognitive tests examined was significantly worse in tAD and PCA patients compared to controls. Compared to tAD patients, PCA patients performed worse on shape detection, object decision, fragmented letters, dot-counting and letter cancellation. PCA patients also performed worse on matrix reasoning relative to tAD patients, likely arising from prominent visual task demands. In contrast, tAD patients performed worse on a verbal recognition memory measure (sRMT words).

**Table 1:** Means, standard deviations, proportions and statistical comparison of demographic, clinical and neuropsychological data for participants included in analysis. For continuous characteristics a Wilcoxon rank sum test was used, while categorical characteristics were compared between groups using Fisher's exact test.

	Participant groups			p-value		
	HC (*n* = 24)	tAD (*n* = 27)	PCA (*n* = 12)	tAD vs HC	PCA vs HC	tAD vs PCA
Demographics & clinical						
Age – years	60.1 (5.7)	61.1 (5.1)	61.2 (4.8)	0.72	0.79	0.93
% female	54%	75%	56%	0.57	0.20	0.22
Handedness (Left: Right)	3:21	1:26	1:11	0.26	0.59	0.53
TIV (cm^3^)	1482 (135)	1461 (171)	1480 (146)	0.65	0.97	0.61
Symptom duration (years)	n/a	5.0 (2.8)	4.6 (2.1)	n/a	n/a	0.89
Education (years)	16.7 (3.0)	15.1 (2.9)	15 (2.6)	0.10	0.10	0.8
MMSE (/30)	29.5 (0.7)	19.8 (5.2)	22.7 (5.1)	<0.0001	<0.0001	0.10
% APOE ε4 carriers	n/a	67%	42%	n/a	n/a	0.17

General intellect						
WASI vocabulary (/80)	68.0 (8.8)	53.5 (17.4)	54.8 (19.6)	0.0009	0.0053	0.78
WASI matrices (/32)	26.7 (2.7)	10.6 (7.8)	4.5 (4.6)^*n*=11^	<0.0001	<0.0001	0.017^a^

Digit span						
forwards (max)	7.2 (1.2)	5.6 (1.4)	5.4 (1.4)	0.0002	0.0009	0.77
backwards (max)	5.5 (1.4)	3.2 (1.4)^*n*=26^	2.7 (1.4)	<0.0001	<0.0001	0.32

Episodic memory						
RMT faces (/25)	24.6 (0.9)	20.2 (4.2)	18.3 (4.1)	<0.0001	<0.0001	0.16
RMT words (/25)	24.3 (1.4)	17.1 (2.9)	20.5 (4.4)	<0.0001	0.0008	0.017

Verbal fluency						
Letter (F)	23.3 (5.4)	10.2 (5.5)	11.8 (5.6)^n=11^	0.011	0.0019	0.89
Category (animals)	15.7 (6.1)	9.4 (4.9)	9.6 (2.3)^n=11^	<0.0001	0.0001	0.11

Reading, spelling & arithmetic						
NART: total errors (/50)	37.8 (8.2)	30.7 (10.8)	30.6 (11.1)^*n*=10^	0.012	0.049	0.93
GDST: oral (/30)	26.1 (4.3)	14.8 (9.2)^n=26^	13.5 (.4)^n=11^	<0.0001	0.0002	0.83
GDA: oral (/24)	14.0 (6.5)	3.4 (5.4)^*n*=25^	2.6 (3.5)	<0.0001	<0.0001	0.74

Visual processing						
VOSP – shape detection (/20)	19.4 (0.8)^*n*=23^	18.5 (1.4)^n=26^	16.9 (2.6)^n=11^	0.0082	0.0001	0.02
VOSP – object decision (/20)	18.0 (1.4)	16.1 (3.1)	10.8 (3.9)	<0.0001	<0.0001	0.0003
VOSP – fragmented letters (/20)	19.5 (0.7)^n=23^	13.2 (7.1)^n=26^	7.0 (5.4)^n=11^	<0.0001	<0.0001	0.013
VOSP – dot counting (/10)	9.9 (0.3)	8.4 (2.7)^n=26^	5.5 (3.3)^n=11^	0.0076	<0.0001	0.0084
A cancellation (time – seconds)	21.1 (6.0)	43.0 (21.1)	69.5 (19.8)^n=11^	<0.0001	<0.0001	0.002
A cancellation (total errors)	0.1 (0.3)	0.6 (1.4)^n=26^	3 (3.7) ^n=11^	0.16	0.0008	0.019

Key: HC = Healthy controls; tAD = amnestic led typical Alzheimer's disease; PCA = posterior cortical atrophy; MMSE = Mini-Mental State Examination; WASI = Wechsler Abbreviated Scale of Intelligence; sRMT = Short Recognition Memory Test; NART = National Adult Reading Test; Graded Difficulty Spelling Test (GDST); GDA = Graded Difficulty Arithmetic; VOSP = Visual Object and Space Perception battery. Where data is only available for a subset of participants, the total n is specified for that variable.

### 3.2 Association between hippocampal subfield volumes and phenotype

**Fig. 2:**
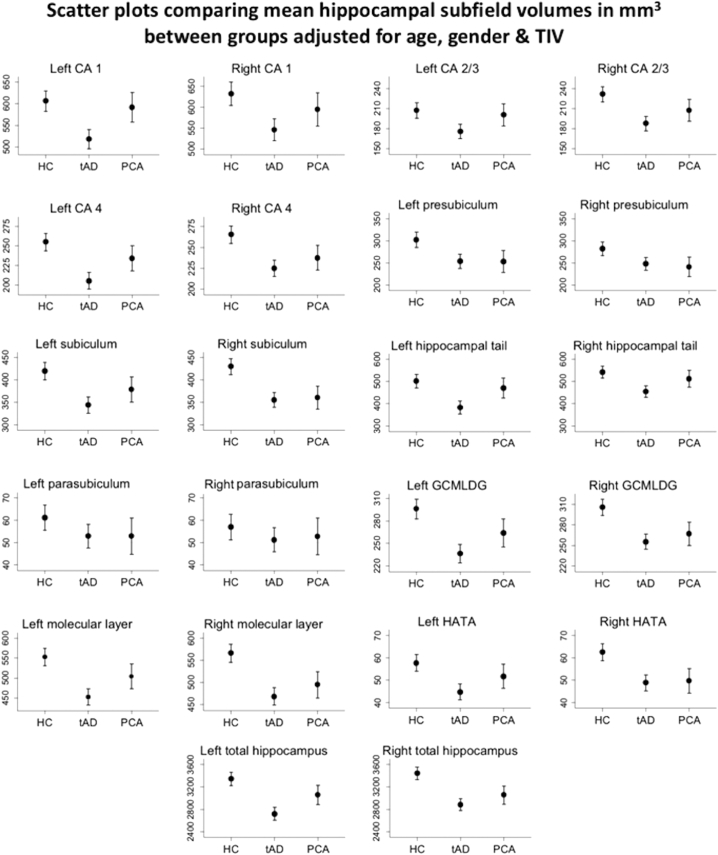
Mean hippocampal subfield volumes in mm^3^ (left and right considered separately) with associated 95% confidence intervals for each participant group. Values are marginal means adjusted for age, sex and TIV following linear regression analysis. Key: HC = Healthy controls; tAD = amnestic led typical Alzheimer's disease; PCA = posterior cortical atrophy; TIV = total intracranial volume; GCMLDG = Molecular and Granule Cell Layers of the Dentate Gyrus; HATA = Hippocampal Amygdala Transition Area.

Unadjusted mean volumes by group for each hippocampal subfield are displayed in Table 2. After adjustment for age, sex and TIV there was strong evidence that tAD patients had widespread volume loss in all hippocampal sub-regions except the parasubiculum (Fig. 2, Table 3).

**Table 2:** Unadjusted mean volumes for each hippocampal subfield by participant group.

	Unadjusted mean volume (SD) (mm^3^)					
	HC		tAD		PCA	
	L	R	L	R	L	R
CA1	613	638	511	541	594	594
	(75)	(83)	(81)	(81)	(97)	(89)
CA2/3	210	234	173	186	202	207
	(38)	(42)	(33)	(30)	(32)	(34)
CA4	257	268	203	223	236	237
	(33)	(38)	(33)	(32)	(32)	(39)
Pre-subiculum	303	284	251	244	257	246
	(41)	(35)	(59)	(55)	(43)	(47)
Subiculum	423	432	341	352	381	364
	(51)	(48)	(58)	(56)	(57)	(46)
Tail	505	546	379	451	474	513
	(69)	(76)	(75)	(65)	(126)	(113)
Para-subiculum	61	57	52	51	54	55
	(13)	(10)	(15)	(18)	(19)	(19)
GCMLDG	298	309	234	253	265	266
	(38)	(42)	(41)	(39)	(36)	(42)
Molecular layer	557	571	447	463	509	496
	(63)	(68)	(76)	(67)	(71)	(68)
HATA	58	63	44	48	52	50
	(12)	(12)	(11)	(12)	(11)	(8)
Total volume	3370	3469	2687	2853	3077	3066
	(367)	(394)	(422)	(393)	(449)	(427)

Key: HC = Healthy controls; tAD = amnestic led typical Alzheimer's disease; PCA = posterior cortical atrophy; GCMLDG = Molecular and Granule Cell Layers of the Dentate Gyrus; HATA = Hippocampal Amygdala Transition Area; L = left; R = right.

In PCA patients compared to controls, after adjustment for age, sex and TIV the strongest evidence for volume loss was in the left presubiclum, right subiculum, right GCMLDG and right molecular layer and the right HATA, (Fig. 2, Table 3).

Comparing EOAD phenotypes, after adjusting for age, sex and TIV, the strongest evidence for decreased volume in tAD compared to PCA was in left CA1 and the left the hippocampal tail (Fig. 2, Table 3).

**Table 3:** Percentage differences in hippocampal subfield volumes (left and right considered separately) between healthy controls, tAD early-onset Alzheimer's disease patients, and PCA early-onset Alzheimer's disease patients estimated using linear regression models (co-variates = age, gender & TIV).

		tAD vs HC		PCA vs HC		tAD vs PCA	
		Mean % difference^a^	p-value	Mean % difference^a^	p-value	Mean % difference^b^	p-value
CA1	L	−15%	<0.001**	−2%	0.49	−12%	0.001**
	R	−13%	<0.001**	−6%	0.12	−8%	0.049*
CA2/3	L	−15%	<0.001**	−3%	0.51	−12%	0.017*
	R	−19%	<0.001**	−10%	0.019*	−10%	0.052
CA4	L	−19%	<0.001**	−8%	0.041*	−12%	0.004*
	R	−15%	<0.001**	−10%	0.004*	−5%	0.17
Pre-subiculum	L	−16%	<0.001**	−16%	0.002**	0%	0.99
	R	−12%	<0.001**	−15%	0.003*	+3%	0.59
Subiculum	L	−18%	<0.001**	−10%	0.019*	−9%	0.045*
	R	−17%	<0.001**	−16%	<0.001**	−1%	0.77
Tail	L	−24%	<0.001**	−6%	0.26	−18%	0.002**
	R	−16%	<0.001**	−5%	0.21	−11%	0.017*
Para-subiculum	L	−13%	0.036	−13%	0.1	0%	0.99
	R	−11%	0.15	−7%	0.41	−3%	0.76
GCMLDG	L	−20%	<0.001**	−11%	0.007*	−11%	0.017*
	R	−16%	<0.001**	−12%	<0.001**	−5%	0.26
Molecular layer	L	−18%	<0.001**	−9%	0.013*	−10%	0.008*
	R	−17%	<0.001**	−12%	<0.001**	−5%	0.15
HATA	L	−22%	<0.001**	−10%	0.076	−13%	0.039*
	R	−22%	<0.001**	−21%	<0.001**	−2%	0.78
Total volume	L	−19%	<0.001**	−9%	0.01*	−11%	0.002**
	R	−16%	<0.001**	−11%	<0.001**	−6%	0.089

Key: TIV = total intracranial volume; GCMLDG = Molecular and Granule Cell Layers of the Dentate Gyrus; HATA = Hippocampal Amygdala Transition Area; PCA = posterior cortical atrophy; tAD = typical Alzheimer's disease; *p ≤.05 – standard statistical threshold; **p **≤** **0.0025** – Bonferroni corrected threshold;

a Expressed as percentage of mean unadjusted volume for relevant subfield in healthy control participants;

b Expressed as percentage of mean unadjusted volume for relevant subfield in PCA patients.

There was no evidence that *APOE* genotype significantly influenced hippocampal subfield volumes in the EOAD patients included in this analysis (see supplementary material).

## 4 Discussion

This study provides evidence for differences in hippocampal subfield volumes in patients with different phenotypic presentations of EOAD. While patients with tAD showed widespread and symmetrical hippocampal volume loss, those with a visual-led PCA phenotype showed relative sparing of hippocampal volume, in line with previous studies (Manning et al., 2015; Peng et al., 2016). In particular, we report evidence of relative preservation of left CA1 and the left hippocampal tail (a conglomeration of CA1-4 and dentate gyrus) volume when directly comparing tAD and PCA phenotypes, providing evidence for differential hippocampal subfield volume in different forms of EOAD.

While this is to our knowledge the first study to assess hippocampal subfields in syndromic variants of Alzheimer's disease, previous hippocampal subfield MRI analysis have found preferential CA1 volume loss in tAD (Apostolova et al., 2010; Blanken et al., 2017; Iglesias et al., 2015; Kerchner et al., 2014; La Joie et al., 2013; Mueller et al., 2010; Pini et al., 2016; Wisse et al., 2014a, Wisse et al., 2014b). Human post-mortem neuropathological studies of tAD (Akram et al., 2008; Price et al., 2001; Scheff et al., 2007; West et al., 1994), as well as animal models of Alzheimer's disease (Helboe et al., 2017; Yang et al., 2018) have also demonstrated early involvement of CA1. CA1 volume loss has previously been shown to be influenced by *APOE* genotype, with the e4 allele being associated with selective CA1 atrophy (Kerchner et al., 2014), however this was not the case in our analysis suggesting *APOE* does not mediate hippocampal subfield volume differences between EOAD phenotypes.

CA1 has been implicated in a range of functions typically impaired in tAD including: autobiographical memory (Bartsch et al., 2011), topographical memory (Bartsch et al., 2010), as well supporting context dependent memory retrieval (Dimsdale-Zucker et al., 2018). One implication of the results of this study is that – in line with our hypotheses – differences in atrophy profiles in individual hippocampal subfields between tAD and PCA may also reflect differences in phenotype, most notably sparing of episodic memory. Future work investigating the relationships between hippocampal subfield volumes and different aspects of episodic memory, for example tasks utilising cues at encoding and retrieval, assessed over different delay conditions (Bird and Luszcz, 1991; Liang et al., 2016), would be of considerable interest, providing *in vivo* insights into subfield mediation of memory subcomponents.

There is a growing body of work suggesting that, while symmetrical hippocampal volume loss is typically considered to be a hallmark of Alzheimer's disease, there is hippocampal asymmetry with evidence that the left hemisphere is subtly more affected than the right in tAD (Barnes et al., 2005; Shi et al., 2009; Wachinger et al., 2016). In this study, we found that, as hypothesised, in PCA there were a greater number of subfields that showed volume loss in the right hippocampus compared to the left hippocampus. This is supported by previous work that has suggested cerebral atrophy in PCA has a right sided predominance compared to tAD (Alves et al., 2013; Lehmann et al., 2011; Whitwell et al., 2018, 2007), but extends it to show that this is also the case for hippocampal subfields. Furthermore, relative preservation of the left hippocampus in PCA compared to tAD has been reported in studies specifically looking at hippocampal volume in loss in PCA (Alves et al., 2013; Manning et al., 2015). However, this right sided predominance was not seen by another study that compared hippocampal volumes between tAD and PCA (Peng et al., 2016), which may reflect different proportions of tAD patients included in each analysis, different disease durations at time of assessment or different statistical approaches. Further work investigating to what extent atrophy in PCA is asymmetrical compared to tAD using more specific markers of asymmetry (Wachinger et al., 2016) will be of interest to explore this further.

Although left CA1 and left hippocampal tail were the only regions to show statistically significance differences between tAD and PCA phenotypes at a strict Bonferroni corrected threshold, many of the subfields (especially within the left hippocampus) showed trends to being spared in PCA relative to tAD. However, one histologically distinct grey matter region that was notable for being involved to an equal extent in both tAD and PCA was the presubiculum. There is evidence from animal studies to suggest that the presubiculum may be a critical hub that integrates information from a wide range of neocortical structures (Insausti et al., 2017). Relevant to PCA, there is evidence that the presubiculum receives direct projections from parietal and occipital cortical regions (Cavada and Goldman-Rakic, 1989; Ding et al., 2000; Seltzer and Pandya, 1984; Seltzer and Van Hoesen, 1979; Vogt and Miller, 1983) and it is possible that the presubiculum volume loss in the PCA patients may reflect disease spread from the occipito-parietal regions to the presubiculm via these afferent connections. This is supported by the current imaging literature, which demonstrates that grey matter atrophy (Lehmann et al., 2011; Poulakis et al., 2018; Ridgway et al., 2012; Whitwell et al., 2018, 2007), glucose hypometabolism (Lehmann et al., 2013; Whitwell et al., 2018), cerebral blood flow (Lehmann et al., 2016) and tau positron emission tomography tracer uptake (Ossenkoppele et al., 2016; Whitwell et al., 2018) are more prominent in occipito-parietal regions in PCA phenotypes. In the case of tAD, the presubiculum has been shown to have reciprocal connections with medial temporal areas (e.g. the entorhinal cortex) (Ding, 2013), potentially making it a similarly vulnerable site in tAD to disease spread from the entorhinal cortex (Carlesimo et al., 2015). Differential patterns of disease spread is an important emerging concept in neurodegenerative disease (Warren et al., 2013) and disease progression modelling techniques (Oxtoby et al., 2017), longitudinal analysis (Iglesias et al., 2016), high resolution connectome imaging (Shi and Toga, 2017), as well as detailed neuropathological study at post-mortem, may provide alternative but complementary avenues to investigate how sequences of disease processes differ or converge across the phenotypic spectrum.

This study has a number of strengths and weaknesses. Firstly, no patients with the more common LOAD were considered in this analysis. While patients with EOAD may not be representative of individuals with LOAD (Dickerson et al., 2017; Joubert et al., 2016; Mendez, 2012), the more variable presentation of EOAD (Rossor et al., 2010) provides an opportunity to explore phenotypic variation. Patients with EOAD are also less likely to have undergone pathological change related to other processes such as ageing and cerebrovascular disease, both of which have been shown to influence hippocampal subfield loss (Mueller and Weiner, 2009; Wisse et al., 2014a, Wisse et al., 2014b; Wu et al., 2008), thus reducing the likelihood that such factors could confound analyses. We used the Freesurfer version 6.0 hippocampal subfield segmentation algorithm (Iglesias et al., 2015). This is based on a high-resolution post-mortem template and addresses concerns regarding previous versions of the software (Wisse et al., 2014a, Wisse et al., 2014b). It is fully automated, eliminating inter-rater variability effects. Furthermore, it is publicly available, which increases scope for replication and comparison of findings between studies. A limitation of the study is the spatial resolution provided by 3T T1-weighted MRI. Although each segmentation was visually checked for obvious errors, precise visualisation of the boundaries that define the distinct hippocampal subfields is not possible at the resolution achievable within a reasonable scan time at this field strength. In particular, results from small volume hippocampal subfields (<100 mm^3^ – i.e. the HATA and parasubiculum) or particularly thin regions (e.g. the molecular layer) may be more difficult to resolve with T1-weighted contrast and could be particularly prone to error. Studies acquiring T1- and T2-weighted images with higher resolution at 7T, investigating hippocampal subfields in Alzheimer's disease, are being undertaken (Blanken et al., 2017; Wisse et al., 2014a, Wisse et al., 2014b); similar studies investigating the differences between PCA and tAD patients using both manual and automated techniques will be important to validate the findings of this study. Furthermore, correlation with detailed neuropathological analysis at post-mortem will be of significant interest. A further limitation of the study is the relatively small sample size, which may limit the ability to identify more subtle differences in some hippocampal subfields. This was particularly the case when considering PCA where only 12 patients with adequate imaging data were available for analysis. As highlighted earlier in the discussion, whilst our findings of more atrophy in left CA1 and worse performance on the RMT (words) in tAD compared to PCA provide indirect evidence implicating left CA1 in the episodic memory impairment that typifies tAD, future work using large enough sample sizes and more comprehensive tests of episodic memory will be required to directly assess the relationships between hippocampal subfield volumes and subcomponents of episodic memory thought to be sub served by individual subfields.

## 5 Conclusion

In summary, these data provide evidence for differential associations between hippocampal subfield volumes and phenotype in EOAD, highlighting areas where atrophy is seen in both tAD and PCA (e.g. the presubiculum) and those where there is relatively sparing of hippocampal subfield volume loss (most notably in left CA1 and the left hippocampal tail) in patients with PCA.

## Declaration of interest

Catherine F. Slattery reports having received personal fees from GE Healthcare. Nick *C*. fox reports personal fees from Janssen/Pfizer, IXICO, Roche, Lilly Research Laboratories (Avid), Novartis Pharma AG, Sanofi and GSK (all fees paid to University College London). Jonathan M. Schott has received research funding from AVID Radiopharmaceuticals (a wholly owned subsidiary of Eli Lilly), has consulted for Roche, Eli Lilly, Biogen and MSD, received royalties from Oxford University Press, given education lectures sponsored by Eli Lilly, and serves on a Data Safety Monitoring Committee for Axon Neuroscience SE. The remaining authors declared no conflicts of interest.

## Appendix A Supplementary data

Influence of APOE genotype on hippocampal subfield volumes in EOAD patients. Key: APOE ε4 +ve = APOE ε4 positive; APOE ε4 -ve = APOE ε4 negative; tAD=amnestic led typical Alzheimer’s disease; PCA=posterior cortical atrophy; TIV=total intracranial volume; GCMLDG=Molecular and Granule Cell Layers of the Dentate Gyrus; HATA=Hippocampal Amygdala Transition Area; † - expressed as percentage of mean volume for relevant subfield in APOE ε4 negative; *p=<0.05 – standard statistical threshold; **p=<0.0025 - Bonferroni corrected threshold Image 1
