# Supplementary material for: Differences in hippocampal subfield volume are seen in phenotypic variants of early onset Alzheimer's disease
Source: Neuroimage Clin. 2018 Dec 11;21:101632. doi: 10.1016/j.nicl.2018.101632 (PMC6411912; doi:10.1016/j.nicl.2018.101632)
Supplement: Supplementary file 1 — Influence of APOE genotype on hippocampal subfield volumes in EOAD patients. Key: APOE ε4 +ve = APOE ε4 positive; APOE ε4 -ve = APOE ε4 negative; tAD=amnestic led typical Alzheimer’s disease; PCA=posterior cortical atrophy; TIV=total intracranial volume; GCMLDG=Molecular and Granule Cell Layers of the Dentate Gyrus; HATA=Hippocampal Amygdala Transition Area; † - expressed as percentage of mean volume for relevant subfield in APOE ε4 negative; *p=<0.05 – standard statistical threshold; **p=<0.0025 - Bonferroni corrected threshold [file mmc1.docx]

|  | Mean unadjusted volume (SD) (mm^3^) | | | | Percentage decrease† following adjustment for TIV, age and gender (95% confidence intervals; p-value) | | | |
| --- | --- | --- | --- | --- | --- | --- | --- | --- |
|  | APOE ε4 -ve (n=16) | | APOE ε4 +ve (n=23) | | APOE ε4 +ve vs -ve  (irrespective of phenotype) | | APOE ε4 +ve vs -ve  (adjusted for phenotype) | |
| Hemisphere | L | R | L | R | L | R | L | R |
| CA1 | 549 (91) | 566 (80) | 527  (95) | 551 (91) | -5%  (-14% to 3% p=0.22) | -3%  (-11% to 5% p=0.41) | -2%  (-10% to 6% p=0.62) | -1%  (-9% to 7% p=0.77) |
| CA2/3 | 184  (31) | 199 (28) | 217  (29) | 188 (35) | -3%  (-14% to 9% p=0.62) | -6%  (-14% to 2% p=0.13) | 1%  (-10% to 12% p=0.88) | -4%  (-11% to 4% p=0.34) |
| CA4 | 217  (29) | 235 (30) | 211 (41) | 222  (36) | -4%  (-14% to 5% p=0.37) | -7%  (-12% to -1% p=0.026*) | -1%  (-10% to 82% p=0.83) | -5%  (-11% to 0% p=0.068) |
| Pre-subiculum | 252 (61) | 238  (52) | 254 (50) | 250 (53) | -3%  (-16% to 9% p=0.58) | 2%  (-9% to 12% p=0.74) | -2%  (-17% to 13% p=0.76) | 1%  (-10% to 12% p=0.88) |
| Subiculum | 363 (58) | 364 (57) | 346 (62) | 350 (50) | -6%  (-15% to 3% p=0.18) | -5%  (-11% to 2% p=0.17) | -4%  (-13% to 5% p=0.36) | -5%  (-12% to 2% p=0.17) |
| Tail | 389 (77) | 468  (92) | 421 (116) | 470  (84) | 5%  (-12% to 21% p=0.57) | -2%  (-12% to 8% p=0.67) | 11%  (-3% to 26% p=0.13) | 1%  (-9% to 11% p=0.81) |
| Para-subiculum | 49 (15) | 45 (11) | 56 (17) | 57  (20) | 6%  (-14% to 26% p=0.52) | 18%  (-4% to 39% p=0.11) | 6%  (-15% to 27% p=0.55) | 19%  (-4% to 42% p=0.095) |
| GCMLDG | 249 (36) | 268 (33) | 240  (46) | 250 (44) | -5%  (-14% to 4% p=0.28) | -7%  (-13% to -2% p=0.011*) | -2%  (-12% to 7% p=0.61) | -7%  (-12% to -1% p=0.027*) |
| Molecular layer | 476 (77) | 483  (62) | 458 (81) | 467 (73) | -6%  (-15% to 3% p=0.17) | -4%  (-10% to 2% p=0.15) | -3%  (-12% to 5% p=0.42) | -3%  (-9% to 3% p=0.29) |
| HATA | 46 (14) | 48  (9) | 46 (10) | 49 (12) | -1%  (-15% to 13% p=0.84) | 0%  (-12% to 12% p=0.96) | 3%  (-11% to 16% p=0.72) | 0%  (-13% to 13% p=0.97) |
| Total volume | 2836 (415) | 2959  (381) | 2788  (500) | 2890  (436) | -4%  (-12% to 5% p=0.37) | -4%  (-9% to 2% p=0.19) | -1%  (-9% to 7% p=0.83) | -2%  (-8% to 3% p=0.41) |

Supplementary material. Influence of APOE genotype on hippocampal subfield volumes in EOAD patients. Key: APOE ε4 +ve = APOE ε4 positive; APOE ε4 -ve = APOE ε4 negative; tAD=amnestic led typical Alzheimer’s disease; PCA=posterior cortical atrophy; TIV=total intracranial volume; GCMLDG=Molecular and Granule Cell Layers of the Dentate Gyrus; HATA=Hippocampal Amygdala Transition Area; † - expressed as percentage of mean volume for relevant subfield in APOE ε4 negative; *p=<0.05 – standard statistical threshold; **p=<0.0025 - Bonferroni corrected threshold
